# Angiogenin and Copper Crossing in Wound Healing

**DOI:** 10.3390/ijms221910704

**Published:** 2021-10-02

**Authors:** Lorena Maria Cucci, Cristina Satriano, Tiziano Marzo, Diego La Mendola

**Affiliations:** 1 Nano Hybrid BioInterfaces Lab. (NHBIL), Department of Chemical Sciences, University of Catania, Viale Andrea Doria 6, 95125 Catania, Italy; lorena.cucci@unict.it; 2 Department of Pharmacy, University of Pisa, Via Bonanno Pisano 6, 56126 Pisa, Italy; tiziano.marzo@unipi.it

**Keywords:** angiogenesis, trophic factor, ribonuclease, cell proliferation, protein, mimicking peptides, copper complexes

## Abstract

Angiogenesis plays a key role in the wound healing process, involving the migration, growth, and differentiation of endothelial cells. Angiogenesis is controlled by a strict balance of different factors, and among these, the angiogenin protein plays a relevant role. Angiogenin is a secreted protein member of the ribonuclease superfamily that is taken up by cells and translocated to the nucleus when the process of blood vessel formation has to be promoted. However, the chemical signaling that activates the protein, normally present in the plasma, and the transport pathways through which the protein enters the cell are still largely unclear. Copper is also an angiogenic factor that regulates angiogenin expression and participates in the activation of common signaling pathways. The interaction between angiogenin and copper could be a relevant mechanism in regulating the formation of new blood vessel pathways and paving the way to the development of new drugs for chronic non-healing wounds.

## 1. Introduction

**Figure 1:**
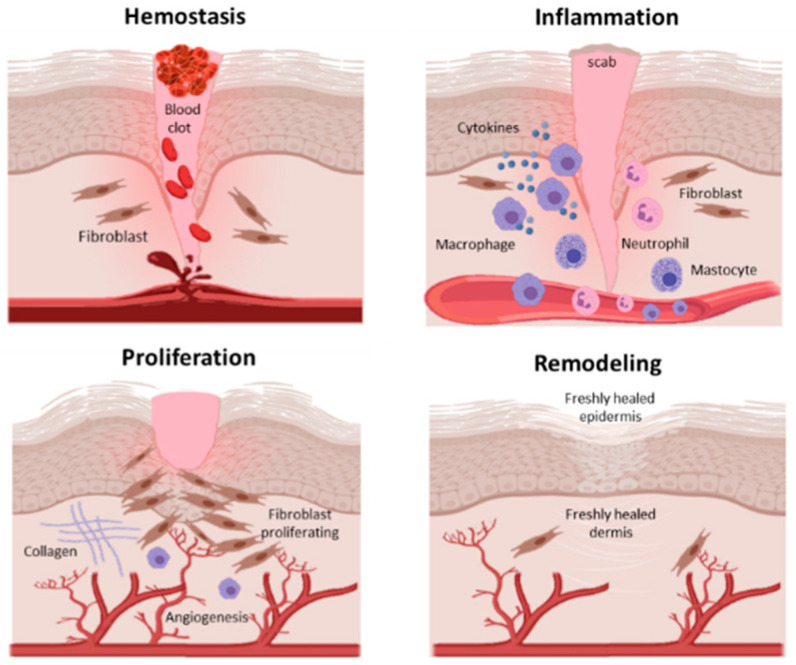
Phases of the wound healing process: hemostasis; inflammation; proliferation; remodeling.

Wound healing is a complex, dynamic and finely balanced series of events starting immediately after an injury. This process includes a strictly coordinated interaction of blood cells, proteins, growth/trophic factors, proteases and extracellular matrix components [1], which takes place in four overlapping phases: (1) hemostasis; (2) inflammation; (3) proliferation; (4) remodeling [2] (Figure 1).

Hemostasis involves the control of blood loss in the damaged region by the activation of the clotting cascade and formation of a large temporary fibrin mesh that fills the wound, preventing microorganism invasion and providing a temporary matrix that serves as a scaffold structure for further healing processes [3].

After hemostasis is achieved, there is a cellular inflammatory response that plays a protective role against invading agents and supports the removal of cell debris. During the inflammatory stage, indeed, immune cells, namely mastocytes, which release cytokines as well as lysosomal enzymes and reactive oxygen species (ROS), migrate to the injury site, thus causing the appearance of oedema and erythema [4]. This process involves also different players, such as Langerhans cells, one of the primary cell types of the immunological barrier, which play an important role during the inflammatory phase of acute wound healing [5], and gamma-delta cells involved in inflammation and re-epithelialization and required for efficient skin wound healing [6,7]

Cell proliferation requires an adequate blood supply in order to receive sufficient oxygen and nutrients [8]; therefore, the following stage is marked by epithelialization, angiogenesis, granulation tissue formation and collagen deposition [1]. This process involves several cell types, including fibroblasts, macrophages and endothelial cells, which exert interdependent activity during wound management. In particular, macrophages provide cytokines that are essential to promote both fibroplasia and angiogenesis; fibroblasts construct the extracellular matrix (ECM), which is indispensable to support the growing tissue; endothelial cells digest and penetrate the vascular basement membrane, invade the ECM and form tube-like structures, which continue to extend branches, thus creating new blood vessel networks [9]. The angiogenic response is stimulated by the release of several soluble molecules capable of regulating cell interactions and stimulating wound angiogenesis. Among them, fibroblast growth factor (FGF) [10], vascular endothelial growth factor (VEGF) [11], platelet-derived growth factor (PDGF), thrombospondin and angiogenin (ANG) play a pivotal role [12,13].

The last step of the proliferation phase consists of fibroblasts’ migration to the wound site and proliferation within the wound for granulation tissue formation. Remodeling is the last phase of wound healing, and at this stage, the maturation of the wound begins. Extracellular matrix components are partially subject to modifications; in particular, collagen type III is replaced by stronger collagen type I, to form a more organized extracellular structure. Fibroblasts and keratinocytes are two cell lines strictly involved in this process, and their interaction, via a paracrine loop, is essential to the outcome of successful dermal remodeling and the transition from granulation tissue to scar formation [14].

Taking into account the complexity and interconnection of the molecular mechanisms involved and the time required for the wound healing process to be fully completed, it is clear that any serious imbalance in the wound healing phases can lead to dysfunction, such as excessive wound healing or chronic wound formation [15,16].

The pathogenesis of excessive wound healing is not fully understood. It is an abnormal form characterized by continuous, localized inflammation in the wound region, which results in excessive collagen synthesis and an overstated accumulation of extracellular matrix components in these wounds. Examples of excessive wound healing are keloid and hypertrophic scars [17].

Keloid scars are characterized by their extensive growth beyond the borders of the original wound, whereas hypertrophic scars are defined as visible and elevated scars that do not spread into surrounding tissues. The precise reason that wound healing sometimes leads to keloid or hypertrophic scar formation is not yet fully elucidated. Both scar types may be uncomfortable but are generally harmless to individual health [18,19,20]. In contrast, pathologies linked to an unhealed wound may be more dangerous.

A wound that has failed to heal in four weeks is defined as a chronic wound. Generally, it is associated with underlying pathologies of a more diverse nature, such as cancer, malnutrition or vascular pathologies. Today, diabetes represents a pathology for which chronic wound healing is a major concern. Diabetic foot ulcers are among the most common complications of patients who have diabetes mellitus and precede the vast majority of amputations in this patient population [21].

Therefore, in diabetes and vascular pathologies, wound care has become increasingly relevant given the rise in chronic wounds and the morbidity associated with them.

A common side effect of injured skin is the possible infection, and many wound dressings have been developed in order to further protect the healing wound from infection and to promote the wound healing process itself.

In this review, we will focus on the role of the angiogenin protein, a potent endogenous angiogenic factor that exhibits antimicrobial properties and an ability to bind copper ions [22,23,24]. Copper is also a known angiogenic factor that regulates different steps of the wound healing process, including the expression of VEGF and ANG [25,26]. Therefore, the potential interconnection between ANG and copper in wound healing processes will be discussed.

## 2. Angiogenin Structure

**Figure 2:**
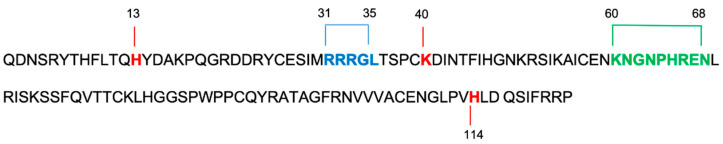
Amino acid sequence of angiogenin using one-letter symbols. The first residue Q, glutamine, is cyclized as pyroglutamate. Red indicates amino acids of catalytic sites (H, histidine; K, lysine); blue indicates nuclear translocation sequence (R, arginine; G, glycine; L, leucine); green indicates cellular binding site (K, lysine, N, asparagine; G, glycine, P, proline, H, histidine; R, arginine; E, glutamic acid).

ANG is a 14,200 Da basic single-chain protein, a member of the RNase family, and is physiologically present in the blood plasma at concentrations of 200–400 ng/mL [27]. It was first discovered and characterized by Vallee and colleagues in 1985 [28]. Its primary structure includes 123 amino acids, with 33% sequence identity and 65% sequence homology with respect to pancreatic ribonuclease A (RNase A). Similarly to RNase A, ANG shows a “kidney-shaped” structure consisting of three α-helices and seven β-sheets stabilized by three disulphide bridges involving the residues Cys-26-Cys-8, Cys-39-Cys-82 and Cys-57-Cys-107, respectively [29]. ANG, as well as RNase A, contains both a catalytic center, formed by the triad His-13, Lys 40 and His-114 (Figure 1), and a purine/pyrimidine binding site [22]. Despite these analogies, the enzymatic activity of ANG is 10^5^–10^6^-fold lower compared to the ribonucleolytic activity of RNase A. The rationale behind the low enzymatic activity of ANG is the obstruction of its pyrimidine base binding site by the glutamine residue, Gln-117, which forms two hydrogen bonds with the threonine residue, Thr-44 [30]. The position of Gln-117 is also determined by intramolecular hydrophobic interactions, which involve the amino acid residues isoleucine, Ile-1 and Ile-19, and phenylalanine, Phe-120 [31]. Accordingly, mutation of the Gln-117 residue leads to an increase in the enzymatic activity of ANG [30]. Furthermore, ANG lacks the fourth disulphide bridge, with respect to RNase A, resulting in the formation of a loop region, including the amino acid sequence 60–68, which is involved in the endothelial cell surface receptor interaction [32]. The ANG structure comprises, also, a nuclear localization sequence encompassing the amino acid sequence 30–35 (Figure 2) [33].

**Figure 3:**
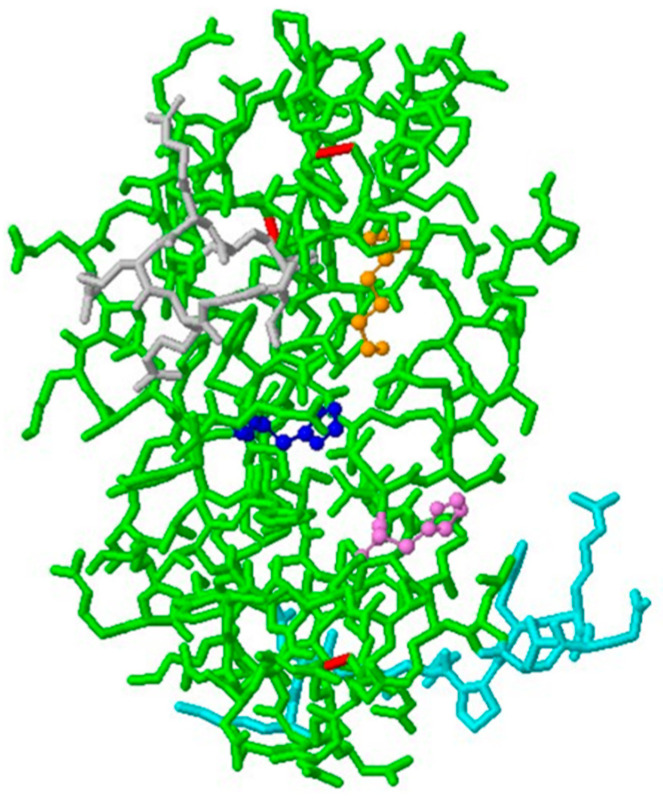
Three-dimensional biological structure of human ANG, consisting of: three disulphide bridges (in red); a catalytic site formed by the triad His-13 (in blue), Lys-40 (in orange) and His-114 (in violet); the receptor binding site, sequence 60–68 (in cyan); the nuclear translocation sequence, 30–35 (in grey). (PDB ID 1ANG, 10.2210/pdb1ANG/pdb).

The catalytic site, the receptor binding site and the nuclear translocation sequence constitute the three characteristic functional sites of ANG, which explain its unique RNase activity and allow for its distinctive biological functions, as a key factor in blood vessel formation [34] (Figure 3).

Interestingly, the integrity of all three functional sites is essential for the maintenance of the biological activity of ANG, since it has been demonstrated that mutation of the His-114 residue causes the complete loss of both the enzymatic activity of the protein and its angiogenic action [24].

### 2.1. Angiogenin Activity

The angiogenic action of ANG is based on protein–protein interactions through which angiogenin promotes endothelial cells’ growth, survival, migration and invasion. Based on data from the literature, ANG binds to a 170 kDa transmembrane receptor located on the surface of angiogenin-responsive endothelial cells. The interaction between ANG and its receptor induces conformational changes in the protein, moving the glutamine Gln-117 residue from its obstructive position and allowing ANG to bind to its natural substrate RNA, and triggers several signal transduction pathways, through the activation of secondary messaging cascades [35].

Recently, plexin-B2 has been identified as a functional angiogenin binding receptor on activated endothelial cells [36].

As result of protein cellular recognition, ANG stimulates signaling pathways related to extracellular signal-regulated kinase 1/2 (ERK1/2) [37], serine/threonine-protein kinases (B/Akt) [38] and stress-associated protein kinase/c-Jun N-terminal kinase (SAPK/JNK) [39], increasing the production of intracellular ribosomal proteins and enhancing cell growth and proliferation. In addition, through the activation of the phosphatidylinositol-3 kinase/Akt pathway (PI3K/Akt), ANG promotes the synthesis and release of nitric oxide, a vasodilator factor, involved in the vascular physiology [40]. Along with the activation of extracellular transduction pathways, ANG is able to pass through the cell membrane, via “receptor-mediated endocytosis”, and accumulates into the nucleus or cytoplasm of endothelial cells, under growth or stress conditions, respectively. Data reveal that inside the nucleus, ANG promotes ribosomal DNA (rDNA) transcription, thus increasing the 47S ribosomal (rRNA) levels by binding to the angiogenin binding element (ABE) on the rDNA promoter, where angiogenin induces methylation and histone modification [41]. Furthermore, ANG enhances the messenger RNA (mRNA) transcription of several genes, since it acts as a chromatin remodeling activator. In this regard, chromatin immunoprecipitation chip assays identified 699 genes that could be regulated by nuclear ANG and most of these genes are significantly expressed in tumorigenesis [42]. On the other hand, the cytoplasmic ANG, following stress conditions (e.g., oxidative damage and starvation stress), cleaves transfer RNA (tRNA) molecules, leading to the production of stress-induced tRNA-derived (tiRNA) [43] molecules, which guide protein translation, thereby promoting damage repair and cell survival.

**Figure 4:**
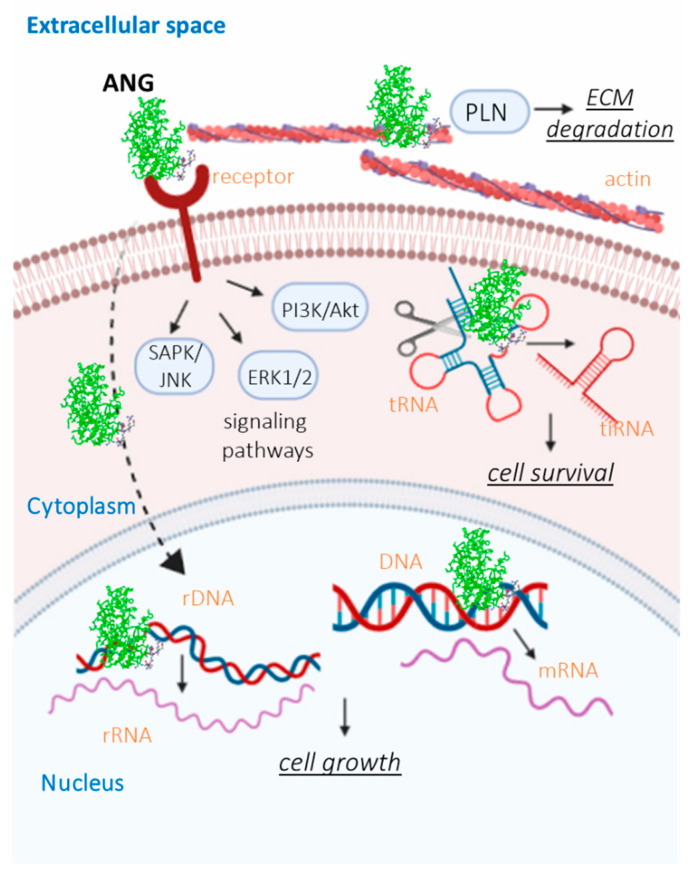
Mechanism of action of ANG. Extracellular ANG, through interaction with an endothelial surface cell receptor, activates several signal transduction pathways, including ERK1/2, SAPK/JNK and PI3K/Akt, thus promoting cell growth and differentiation. Moreover, extracellular ANG undergoes receptor-mediated endocytosis and accumulates in the cytoplasm, under stress conditions or in the nucleus, under growth conditions. Nuclear ANG stimulates both rRNA and mRNA transcription, allowing for cellular growth. In addition, ANG interacts with cell surface actin, leading to ECM and basement membrane degradation, thereby promoting cell migration and invasion.

It is also known that ANG, through its cell surface receptor binding site, forms a complex with the endothelial cell surface α-actin [44]. The relevance of this protein domain is further confirmed by the mimic role of a peptide encompassing the amino acid sequence 60–68, which interacts with actin in a similar way to the whole ANG protein [45]. α-actin binding is a crucial step for the promotion of angiogenesis since this complex is able to activate the plasminogen activator/plasmin serine protease system, leading to plasmin (PLN) generation from plasminogen [44,46]. Plasmin, indeed, is an enzyme able to degrade both laminin and fibronectin in the basement membrane and the extracellular matrix, thus promoting endothelial cell migration and invasion into the perivascular tissue, which is a crucial phase of vessel growth [47]. Furthermore, upon interaction with actin, ANG induces changes in the cell cytoskeleton by inhibiting the polymerization of G-actin and changing the physical properties of F-actin, respectively [44]. These events severely alter the cells’ mechanical properties, thus inducing strong effects on the cellular structure and function, tissue morphogenesis as well as the whole angiogenic process [22] (Figure 4).

Nevertheless, the widespread expression of ANG in several human tissues and its presence in fluids, namely plasma [48], the tumor microenvironment [49] and amniotic [50] and cerebrospinal liquids [51], suggests its participation not merely in neovascularization but also in further physiological and pathological processes, including neuroprotection [52], inflammation [53], the immune response [54], micro-biocidal activity [55] and reproduction [56]. Accordingly, mutations of the gene encoding for ANG have been found in patients affected by neurodegenerative disorders, such as amyotrophic lateral sclerosis (ALS) [57] and Parkinson’s disease (PD) [58], while an increased concentration of ANG has been measured in patients with ulcerative and Crohn’s diseases (CrD) [59].

### 2.2. Angiogenin and Wound Healing

**Figure 5:**
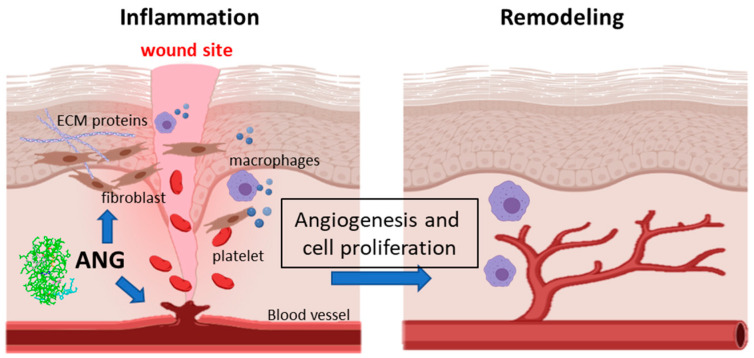
Angiogenin promotes wound healing by inducing angiogenesis and cell migration and by activating fibroblast cells to produce ECM proteins (collagen, fibrin, fibronectin).

As reported above, ANG is directly involved in the wound healing process as its primary biological function is blood vessel homeostasis regulation, through both the stimulation of new vessel growth and the maintenance of endothelial cell self-renewal. Furthermore, ANG activates fibroblasts and the factors that they produce, thus also indirectly influencing the course of wound healing (Figure 5).

The use of angiogenin for the topical treatment of wounds and ulcerous damage in humans has been tested and patented in Russia [60]. This is claimed to reduce the time to wound recovery and tissue generation; however, at present, and to the best of our knowledge, there are no pharmaceutical forms containing ANG for wound healing applications. An effective application requires, among other aspects, a more accurate understanding of the mechanism by which the protein promotes wound repair.

Intracutaneous injections of recombinant angiogenin in Wistar rats induce a dose effect causing morphological changes in the dermis, playing a relevant role in regenerative processes [61]. The thickness of the stratum corneum is enhanced, as well as the density of collagen fibers and the proliferation rate of epidermal cells in animals administrated with ANG compared to those used as controls. In the same work, it is demonstrated that the addition of recombinant ANG stimulates the blood cells to produce and release both pro- and anti-inflammatory cytokines, suggesting that ANG may act as a protective homeostatic factor through angiogenic process activation or through the activation in the dermal blood vessels of other circulating cells, such as lymphocytes, neutrophils and endothelial cells. Therefore, ANG may exert wound healing effects by triggering different and combined biochemical pathways in the basal layer of the dermis.

Wound healing promotion by ANG is observed in different endothelial cells. The corneal endothelial cells (CECs) form the innermost monolayer of the cornea and need to be physiologically protected against injuries [62]. A scratch wound assay carried out on CECs shows that ANG promotes cell migration and wound closure by the activation of the phosphatidylinositol 3-kinase (PI3-k) signaling pathway [63]. In the same study, the healing effect is also observed in an in vivo test. Treatment with ANG eye drops significantly reduced corneal haziness in a rabbit model of transcorneal freezing injury, in which the corneal endothelial layer was destroyed by freezing [63].

The properties of ANG have prompted the development of engineered CECs able to overexpress the protein, with the aim of mimicking the corneal endothelium in vivo and enhancing graft cellularity for transplantation approaches [64].

An important aspect of ANG’s action is its involvement in the innate immune system. The protein is also a component of tears and displays an immune modulatory function in corneal fibroblasts [65]. Experiments carried out on a rat model of corneal alkali burns showed that ANG addition in vivo recovered normal cornel transparency and caused a significant reduction in the corneal opacity score compared to the control [66].

A high level of ANG has been measured in wound fluids collected post-injury [67,68]. On the other hand, it has been demonstrated that high levels of ANG in wound fluids are able to induce endothelial cell proliferation and circular angiogenic cell (CAC) differentiation, while the antibody neutralization of ANG in equivalent wound fluids leads to a reduction in their angiogenic properties [67]. Such findings are also supported by further clinical data and prove the positive correlation between ANG and the wound healing process [69].

## 3. The Role of Copper in Angiogenesis

Copper is an essential element crucial to the health of living organisms [70]. Many studies have highlighted the distinctive biological role of copper ions in both neuronal and endothelial tissues that, despite their different biological structures, share similar signaling pathways [71,72,73]. In the neuronal system, copper ions seem to play a pivotal role in post-synaptic transmission, and its dyshomeostasis is involved in the etiology of numerous neurologic disorders, such as ALS, prion encephalopathies, PD and Alzheimer’s disease [74,75,76,77]. Regarding its interaction with the endothelial system, copper has been recognized as an angiogenic factor. Data from the literature demonstrate that copper ions are able to stimulate endothelial cell migration [78] and neovascularization in avascular rabbit corneas [79], while its depletion, by Cu chelators such as penicillamine and trientine, prevents vessel formation in vivo [80,81]. The role of copper in angiogenesis supports the critical role of the metal in pathological and physiological angiogenic processes, such as cancer and wound repair. Accordingly, increased serum levels of copper have been found in patients with different types of tumors and are related to tumor onset and progression. Based on these findings, copper chelation therapies have been developed for cancer treatment and have proven their efficacy in tumor regression [82].

Later, it was realized that the removal of excess extracellular metal was not in itself a solution [80,81,83]. Copper regulates the expression of different proteins, and in cancer or other pathologies, there are specific metal transporters that are over- or underexpressed. There is a strict balance between pathology and physiology, so the proper and correct restoration of copper homeostasis may represent a valuable pharmacological approach [84].

Indeed, the local modulation of copper’s pro-angiogenic effect provides a promising strategy to enhance tissue repair and regeneration [85] since, as expected, a higher concentration of copper ions (~30 μM) has been detected in the injured site with respect to the peri-wound areas [86].

The mechanism behind the pro-angiogenic activity of copper is the activation and amplification of the angiogenic response, triggered by several cytokines and proteins including VEGF, FGF and ANG, through a multi-faceted action.

The intracellular uptake of copper ions, and their efflux as well as trafficking within the cytoplasmic matrix, is tightly controlled by a complex protein network, since copper shows a reactive nature, which could lead to severe oxidative damage, as long as the free cytosolic copper concentration exceeds the bio-recommended levels (10^−18^ M) [87]. Copper, indeed, can exist either in the reduced state, Cu^+^, which shows a high affinity for the thiol and thioether groups of the proteins, or in the oxidized state, Cu^2+^, which particularly binds to oxygen atoms and imidazole nitrogen. Although its double nature allows copper to interact with several proteins, thus controlling numerous biochemical processes, the passage between the two states, Cu^+^ and Cu^2+^, can generate hydroxyl radicals [88].

Copper enters the cell through the copper transporters 1 and 2 (CTR1 and CTR2), which are membrane proteins with a channel-like structure, widely present in several cell types and tissues [89,90,91]. Before its transfer, to ensure efficient transport across the cell membrane, Cu^2+^ is reduced to Cu^+^, by membrane metal reductases [92], and within the cell, copper’s distribution to mitochondrial, nuclear and vesicular targets is mediated by several metal chaperones, namely glutathione (GSH), Menkes protein, copper chaperone for superoxide dismutase (SOD) and antioxidant-1 (ATOX-1) [93].

During the early stages of angiogenesis, intracellular copper has been demonstrated to stabilize the hypoxia inducible factor-1 (HIF-1) structure, thereby promoting its transcriptional activity on angiogenic genes including VEGF and ceruloplasmin genes [94,95,96]. Ceruloplasmin, indeed, is a serum globulin protein that, by binding to copper ions, stimulates neovascularization, whereas its copper-deprived form is unable to induce blood vessel formation [97]. Regarding VEGF, experiments on cultured human cardiomyocytes showed that copper ions, at a concentration of 5 μM, stimulate insulin-like growth factor-1 (IGF-1)-induced VEGF expression [98]. Moreover, copper ions have been found to promote vasodilation by activating the endothelial nitric oxide synthase enzyme (eNOS) and nitric oxide (NO) release. In this regard, several studies suggest that extracellular Cu^2+^ promotes transmembrane calcium ion influx, increasing the concentration of intracellular Ca^2+^ ions, which, via the calcium-calmodulin pathway, enhances the eNOS activity [99]. On the other hand, intracellular copper, as a cofactor of the cuproenzyme SOD, protects NO from superoxide anion scavenging, thus increasing its half-life [100]. Furthermore, copper complexes of the fibroblast growth factor 1 (FGF-1) and the lysyl oxidase (LOX) [101] stimulate FGF secretion [102] and ECM degradation, respectively, thus promoting cell migration and proliferation. It is important to note that the copper-induced angiogenic effects are highly cell-type-specific, since it has been demonstrated that copper ions are able to enhance the proliferation of endothelial cells, while, under the same conditions, no stimulating effects have been found in arterial smooth muscle cells and even weaker effects have been measured in cultured fibroblasts [103].

## 4. Copper Modulates Angiogenin Activity

Copper ions amplify and promote vascular permeabilization as well as endothelial cells’ migration and proliferation by binding to several factors involved in the angiogenic process. Among the pro-angiogenic effectors, it has been found that copper controls and modulates the angiogenic response and the biological function of ANG.

Copper increases ANG expression in the HUVEC cell line, so it is possible that the increase in extracellular copper during the angiogenesis process may regulate the angiogenin level [26].

Indeed, copper is the only metal mobilized during the angiogenesis process from the intra- to extracellular space [104]. ANG is a secreted protein that translocates inside the cell, so a direct interaction between metal and protein may constitute a system of mutual control between the two components in different angiogenic steps.

Previous works reported that the complex formation between divalent copper and ANG decreases both the nuclear translocation of the protein and its ribonucleolytic activity. Moreover, the interaction between ANG and calf pulmonary artery endothelial cells increases 4.3-fold in the presence of copper ions [105].

**Figure 6:**
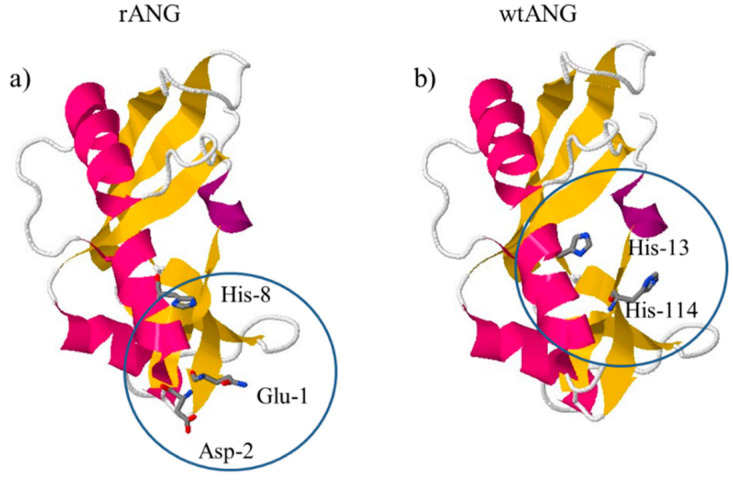
Putative anchor site of Cu^2+^ in (**a**) rANG and (**b**) wtANG copper complexes (PDB ID 1ANG).

The ANG–copper complex formation, the metal coordination environment as well as the copper-induced effect on ANG mostly depend on the chemical structure of the protein, and angiogenin, in particular, shows two different forms, the recombinant and the wild type. The recombinant form of ANG (rANG), expressed in bacterial vectors and typically used for research works, contains an extra methionine residue at the N-terminal domain. Differently, the wild-type angiogenin (wtANG), physiologically present in human plasma, shows glutamine as the first residue, which is spontaneously cyclized to a pyroglutamate ring. A recent research work showed that rANG and wtANG share a similar secondary structure rich in β-strands, but with different metal binding. Circular dichroism (CD) and electrospray ionization mass spectrometry (ESI-MS) experiments, indeed, suggest a 2:1 metal to ligand stoichiometry of the metal–protein complex for rANG, while a 1:1 metal to ligand stoichiometry for the copper complex of wtANG, at physiological pH [24], has been noted. Furthermore, spectroscopic data revealed a strong ligand field around the metal core of the rANG–Cu(II) complex, which involves four nitrogen donors in a planar arrangement [24,106]. Meanwhile, a low ligand field seems to characterize the wtANG–Cu(II) complex, which most likely involves two imidazole nitrogen atoms, one deprotonated nitrogen and one oxygen atom [24,106]. NMR measurements allowed the identification, at physiological pH, of the N-terminal group of the methionine, the deprotonated amide nitrogen of Glu-1 and Asp-2 and the imidazole nitrogen of the His-8 residues in the coordination environment of the rANG–Cu(II) complex. On the other hand, His-114 and His-13, which also form the catalytic site of ANG, are the metal anchoring sites for the formation of the wtANG–Cu(II) complex (Figure 6). Accordingly, copper ions more efficiently influence the physiological form of ANG, with respect to the recombinant angiogenin, since the metal–protein complex involves its catalytic site. In this regard, in vitro experiments of capillary-like tube formation and the RNase enzymatic assay evidenced that divalent copper decreases the activity of both the proteins, but a higher concentration of Cu^2+^ was required for the rANG sample to reach the same decrease in both tube formation and enzymatic action observed for wtANG [24].

The design of peptides able to mimic the functional sites of proteins is an interesting strategy aimed at understanding protein activity and cofactor binding as metal ions as well as in developing new potential drugs [107,108,109]. Peptides encompassing the N-terminal residues 1–17 of the protein, Ang(1–17), with the amino free, and AcAng(1–17), the analogous form with the N-terminal amino group acetylated, were synthesized to highlight the role of the amino group in copper binding [110]. A physiological concentration of copper increases actin staining, an effect counteracted by the addition of copper complexes formed by N-terminal peptides. The comparison between wtANG, rANG and N-terminal peptides highlights that a different copper coordination environment affects its biological activities.

The medical treatment of pathological angiogenesis as well as the modulation of physiological vessel growth, through the use of angiogenin or its peptide fragments, can be improved by using nanocarriers. Experiments carried out on endothelial cells revealed a significant improvement in wound closure and angiogenic activity after treatment with a multifunctional platform obtained by anchoring ANG to the surfaces of AuNPs [111]. Notably, this new hybrid nanoplatform triggers intracellular copper trafficking, confirming the correlation between metal and ANG in the angiogenic process.

## 5. Conclusions

Angiogenin is a secreted protein that regulates different angiogenesis steps by means of multiple pathways, many of which are yet to be fully elucidated. Some of these pathways are also activated by copper, another angiogenic factor. Copper regulates ANG expression in endothelial cells and many experimental studies suggest that metal drives ANG intracellular localization. On the other hand, experiments carried out on cancer cells show that ANG may regulate intracellular copper levels. Therefore, ANG and copper activity in angiogenic processes appear to be closely related and the understanding of their common biochemical pathways is expected to pave the way to new pharmaceutical applications in wound healing.
